# Identification of significant genes associated with prognosis of gastric cancer by bioinformatics analysis

**DOI:** 10.1186/s43046-022-00157-w

**Published:** 2022-12-26

**Authors:** Shuanhu Wang, Song Tao, Yakui Liu, Yi Shi, Mulin Liu

**Affiliations:** Department of Gastrointestinal Surgery, The First Affiliated Hospital of Bengbu Medical College, Bengbu, Anhui China

**Keywords:** Stomach neoplasms, Gene expression profiling, Prognosis, Bioinformatics

## Abstract

**Background:**

Gastric cancer (GC) ranks second in mortality among all malignant diseases worldwide. However, the cause and molecular mechanism underlying gastric cancer are not clear. Here, we used integrated bioinformatics to identify possible key genes and reveal the pathogenesis and prognosis of gastric cancer.

**Methods:**

The gene expression profiles of GSE118916, GSE79973, and GSE29272 were available from the Gene Expression Omnibus (GEO) database. Differentially expressed genes (DEGs) between GC and normal gastric tissues were screened by R software and Venn diagram software. GO and KEGG pathway enrichment of DEGs was performed using the DAVID database. A protein-protein interaction (PPI) network was established by STRING and visualized using Cytoscape software. Then the influence of hub genes on expression and survival was assessed using TCGA database.

**Results:**

A total of 83 DEGs were found in the three datasets, including 41 up-regulated genes and 42 down-regulated genes. These DEGs were mainly enriched in extracellular matrix organization and cell adhesion. The enriched pathways obtained in the KEGG pathway analysis were extracellular matrix (ECM)-receptor interaction and focal adhesion. A PPI network of DEGs was analyzed using the Molecular Complex Detection (MCODE) app of Cytoscape. Four genes were considered hub genes, including COL5A1, FBN1, SPARC, and LUM. Among them, LUM was found to have a significantly worse prognosis based on TCGA database.

**Conclusions:**

We screened DEGs associated with GC by integrated bioinformatics analysis and found one potential biomarker that may be involved in the progress of GC. This hub gene may serve as a guide for further molecular biological experiments.

## Background

Gastric cancer (GC) is the sixth most commonly diagnosed cancer. Its mortality rate places it second among the malignant tumors worldwide [1]. The 5-year overall survival rate of patients in the early stage can reach 95% [2], but for patients in the advanced stage, it has remained at about 50% even after comprehensive treatment based on surgery [3, 4]. The cause of the low survival rate is tumor recurrence and metastasis. Therefore, it is important to study the potential molecular mechanism underlying the malignant biological behavior of GC cells and find effective early diagnostic techniques and reliable molecular markers for monitoring recurrence and evaluating prognosis. Despite major advances in the understanding of the molecular mechanisms of GC and in emerging targeted therapeutic options, not all patients see effective results from existing targeted therapies [5, 6].

In recent years, the use of microarray and RNA-sequencing technology has provided an efficient tool in the search for promising biomarkers for cancer diagnosis, treatment, and prognosis [7, 8]. A large amount of data has been collected on public database platforms such as Gene Expression Omnibus (GEO) and the Cancer Genome Atlas (TCGA). These databases can be used to study the molecular mechanism further. A lot of research has been done on the gene expression profile of GC. The exact molecular mechanism of the GC is far from fully uncovered [9]. There is considerable need to find more potential for effective therapeutic strategies.

In order to better understand the influence of DEGs on molecular pathogenesis of GC, in this study, we downloaded three gene expression profiles from the GEO database and screened DEGs. We performed further gene ontology (GO) and Kyoto Encyclopedia of Genes and Genomes (KEGG) pathway enrichment of DEGs. Finally, key genes affecting the prognosis of GC patients were identified using the PPI network and survival analyses.

## Methods

### Microarray data and identification of DEGs

Three sets of microarrays, GSE118916, GSE79973, and GSE29272, were downloaded from the Gene Expression Omnibus (http://www.ncbi.nlm.nih.gov/geo/) database. We only chose paired GC tissues and their matched adjacent tissues. When multiple probes were found to correspond to one specific gene, the average level of expression was considered to be its final expression. The original microarray data of each series were processed using R software package (version 3.6.1; http://www.R-project.org/). The data were log_2_ transformed. |Log_2_ fold change (FC)| > 1 and adjusted *P* < 0.01 were considered the cutoff criteria for DEG screening. A Venn diagram was created using Venny (version 2.1; https://bioinfogp.cnb.csic.es/tools/venny/index.html). All common DEGs in these three datasets were selected for further study.

### GO and KEGG pathway enrichment analysis

GO is a common method for annotating a large number of genes [10]. KEGG is an integrated database resource for biological interpretation of genome sequences and other high-throughput data [11]. GO and KEGG pathway enrichment analysis was performed using the database for annotation, visualization, and integrated discovery (DAVID) online tool (version DAVID 6.8; http://david.ncifcrf.gov/), which provides a comprehensive set of functional annotation tools for investigators to understand the biological meaning behind the large list of genes [12]. A *P* < 0.05 was considered statistically significant.

### PPI network construction and hub gene identification

The Search Tool for the Retrieval of Interacting Genes (STRING; version 11.0; http://string-db.org/cgi/input.pl) was used to explore the protein-protein interaction (PPI) information of DEGs. Validated interaction score > 0.4 was selected as the cutoff criterion. Cytoscape software (version 3.6.0; http://www.cytoscape.org/) was used to visualize and analyze integration of PPI networks. The Molecular Complex Detection (MCODE) app with default parameters in Cytoscape was used to filter modules of the entire network. The cytoHubba app of the Cytoscape software was used to select important hub genes among these DEGs. We use the density of maximum neighborhood component (DMNC) and maximal clique centrality (MCC) methods provided in the cytoHubba app. Mutual genes from two methods were selected as hub genes.

### Validation and survival analysis based on TCGA database

To validate the results of hub genes, expression on box plots of GC from the Cancer Genome Atlas (TCGA) database was used to show the expression patterns between tumor and normal samples. Survival and stage analysis of the hub genes were also made with the Gene Expression Profiling Interactive Analysis (GEPIA) online database (http://gepia.cancer-pku.cn/detail.php).

## Results

### Microarray data information and identification of DEGs

**Fig. 1:**
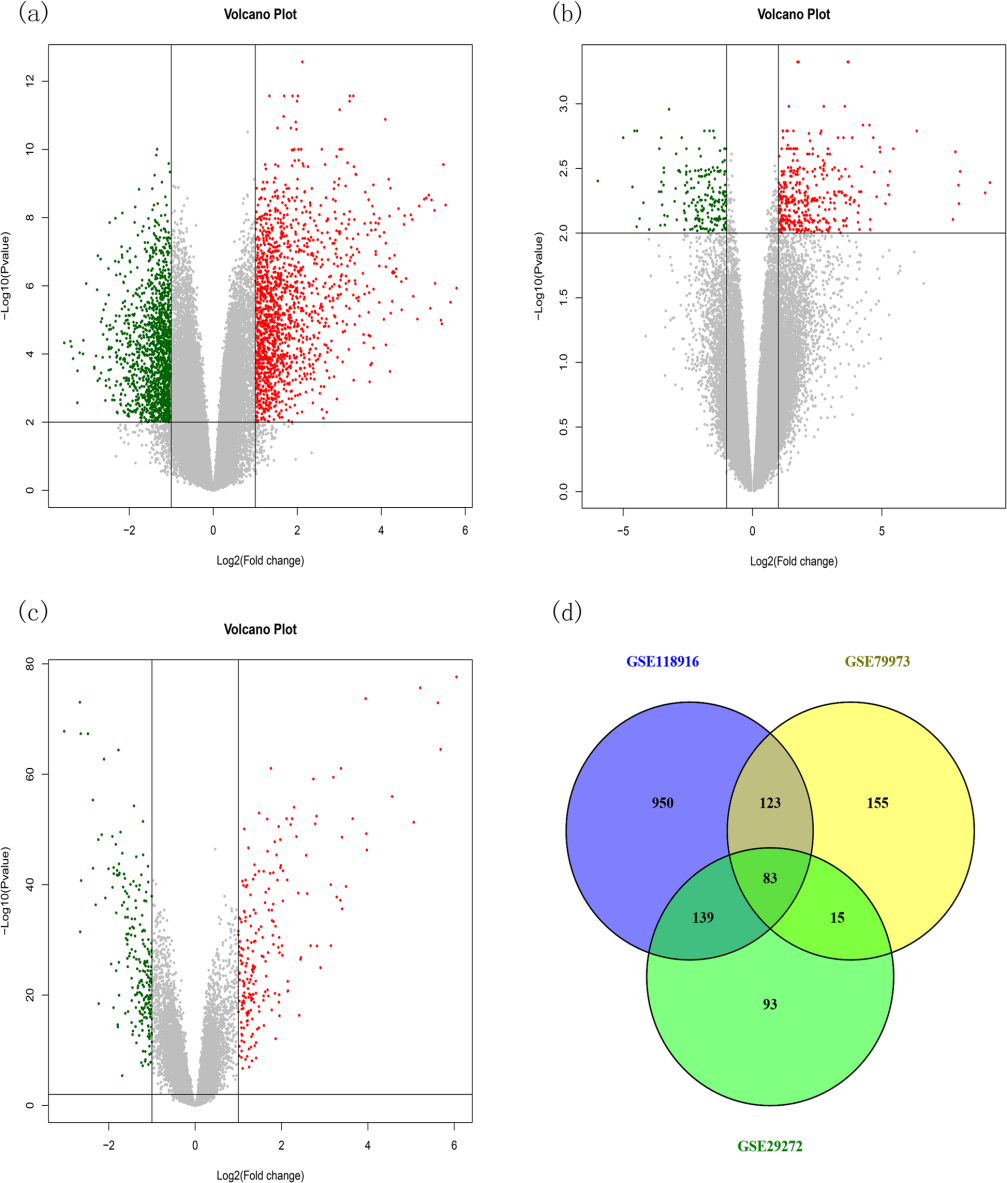
Identification of DEGs among each GEO data set. **a**–**c** The volcano plots of the distribution of DEGs in each data set. **d** Authentication of 83 common DEGs in the three datasets (GSE118916, GSE79973, and GSE29272) through Venn diagram software (available online: https://bioinfogp.cnb.csic.es/tools/venny/index.html)

Three gene expression profiles (GSE118916, GSE79973, and GSE29272) were acquired from GEO database. The detailed information of these three gene expression profiles is shown in Table 1. There were a total of 318 samples, including 159 tumor and 159 matched adjacent tissues. There were 1295 DEGs, including 651 upregulated and 644 downregulated genes, in GSE118916. A total of 376 DEGs were screened from the GSE79973 data set, including 132 upregulated and 244 downregulated genes. Another 330 DEGs were selected from the GSE29272 data set, including 165 upregulated and 165 downregulated genes. The volcano plots of DEGs among each data set are shown in Fig. 1 a–c. A total of 83 genes were screened out in all three datasets for further analysis (Fig. 1d). There were 41 upregulated genes and 42 downregulated genes in GC tissues compared to adjacent tissues (Table 2).

**Table 1:** Information for GEO gastric cancer data

Reference	Dataset ID	Country	Platform	No. of samples (normal/tumor)
Li et al. (2019) [13]	GSE118916	China	GPL15207 [PrimeView] Affymetrix Human Gene Expression Array	15/15
He et al. (2016) [14]	GSE79973	China	GPL570 [HG-U133_Plus_2] Affymetrix Human Genome U133 Plus 2.0 Array	10/10
Wang et al. (2013) [15]	GSE29272	China	GPL96 [HG-U133A] Affymetrix Human Genome U133A Array	134/134

*GEO* Gene Expression Omnibus

**Table 2:** Detected DEGs in gastric cancer by integrated microarray

DEGs	Gene names
Upregulated	AEBP1 ANOS1 APOC1 ASPN BGN CALD1 CDH11 COL10A1 COL18A1 COL1A1 COL1A2 COL3A1 COL4A1 COL5A1 COL5A2 COL6A3 DPYSL3 FBN1 FN1 FSTL1 IGF2BP3 IGFBP7 INHBA LGALS1 LUM MEST NID2 OLFML2B PMEPA1 RAB31 RARRES1 SFRP4 SKAP2 SPARC SPP1 SULF1 THBS1 THBS2 THY1 TIMP1 VCAN
Downregulated	AKR1B10 AKR1C1 ALDH3A1 ALDOB ATP4A ATP4B AZGP1 CAPN9 CCKBR CKMT2 CPA2 CYP2C18 CYP2C9 CYP3A5 DGKD EPB41L4B ESRRG ETNPPL FOLR1 GATA6 GIF GKN1 GPRC5C HMGCS2 HPGD HRASLS2 KCNJ15 KCNJ16 MT1E MT1F MT1G MT1H MT1M MT1X MYRF NEDD4L NQO1 PBLD PLLP PXMP2 TMPRSS2 UBL3

*DEGs* differentially expressed genes

### GO and KEGG pathway enrichment analysis of DEGs

GO and KEGG pathway enrichment of all 83 DEGs was analyzed using the DAVID online tool. The GO enrichment analysis results were divided into three functional categories, biological processes (BP), cell component (CC), and molecular function (MF). In the BP category, the genes were significantly enriched in extracellular matrix organization, collagen catabolic process, and cell adhesion categories. In the CC category, the genes were significantly enriched in extracellular exosome and extracellular regions. In the MF category, the genes were significantly enriched in calcium ion binding and identical protein binding. The details are shown in Table 3. The signaling pathways of DEGs were mainly enriched in extracellular matrix (ECM)-receptor interaction, protein digestion and absorption, focal adhesion, and PI3K-Akt signaling pathway (Table 4).

**Table 3:** GO analysis of DEGs associated with gastric cancer

Term	Description	Count	***p***-value
GO:0030198	Extracellular matrix organization	19	1.34E-18
GO:0030574	Collagen catabolic process	9	6.62E-10
GO:0007155	Cell adhesion	16	3.69E-09
GO:0071294	Cellular response to zinc ion	6	2.39E-08
GO:0045926	Negative regulation of growth	6	2.39E-08
GO:0001501	Skeletal system development	9	2.83E-07
GO:0071276	Cellular response to cadmium ion	5	1.08E-06
GO:0030199	Collagen fibril organization	6	1.10E-06
GO:0031012	Extracellular matrix	20	4.21E-17
GO:0005578	Proteinaceous extracellular matrix	17	5.36E-14
GO:0005576	Extracellular region	30	2.64E-11
GO:0005615	Extracellular space	27	8.90E-11
GO:0005581	Collagen trimer	9	8.13E-09
GO:0070062	Extracellular exosome	34	4.93E-08
GO:0005604	Basement membrane	8	6.42E-08
GO:0005788	Endoplasmic reticulum lumen	10	2.00E-07
GO:0005201	Extracellular matrix structural constituent	11	5.59E-13
GO:0048407	Platelet-derived growth factor binding	5	1.43E-07
GO:0050840	Extracellular matrix binding	5	6.14E-06
GO:0008201	Heparin binding	7	1.04E-04
GO:0005178	Integrin binding	6	1.32E-04
GO:0005518	Collagen binding	5	1.78E-04
GO:0005509	Calcium ion binding	11	0.001830946
GO:0042802	Identical protein binding	10	0.008153182

*GO* gene ontology, *DEGs* differentially expressed genes

**Table 4:** KEGG pathway analysis of DEGs associated with gastric cancer

Pathway ID	Name	Count	***p***-value	Genes
hsa04512	ECM-receptor interaction	11	4.20E-10	COL4A1 COL3A1 COL6A3 COL1A2 COL1A1 THBS1 THBS2 COL5A2 COL5A1 SPP1 FN1 COL18A1 COL4A1 COL3A1 COL6A3 COL1A2 CPA2 COL1A1 COL5A2 COL5A1 COL10A1 COL4A1 COL3A1 COL6A3 COL1A2 COL1A1 THBS1 THBS2 COL5A2 COL5A1 SPP1 FN1 MT1M MT1E MT1H MT1X MT1G MT1F COL4A1 COL3A1 COL1A2 COL1A1 COL5A2 COL5A1 FN1 COL4A1 COL3A1 COL6A3 COL1A2 COL1A1 THBS1 THBS2 COL5A2 COL5A1 SPP1 FN1 KCNJ16 KCNJ15 CCKBR ATP4A ATP4B COL3A1 COL1A2 COL1A1 COL5A2 COL5A1 CYP3A5 CYP2C9 AKR1C1 ALDH3A1 CYP3A5 CYP2C9 CYP2C18 ALDH3A1
hsa04974	Protein digestion and absorption	10	9.88E-09	
hsa04510	Focal adhesion	11	1.68E-06	
hsa04978	Mineral absorption	6	1.45E-05	
hsa05146	Amoebiasis	7	1.06E-04	
hsa04151	PI3K-Akt signaling pathway	11	1.50E-04	
hsa04971	Gastric acid secretion	5	0.001859357	
hsa04611	Platelet activation	5	0.014342191	
hsa00980	Metabolism of xenobiotics by cytochrome P450 Chemical	4	0.016315925	
hsa05204	carcinogenesis	4	0.020062655	

*KEGG* Kyoto Encyclopedia of Genes and Genomes, *DEGs* differentially expressed genes, *ECM* extracellular matrix

### PPI network construction and selection of hub genes

**Fig. 2:**
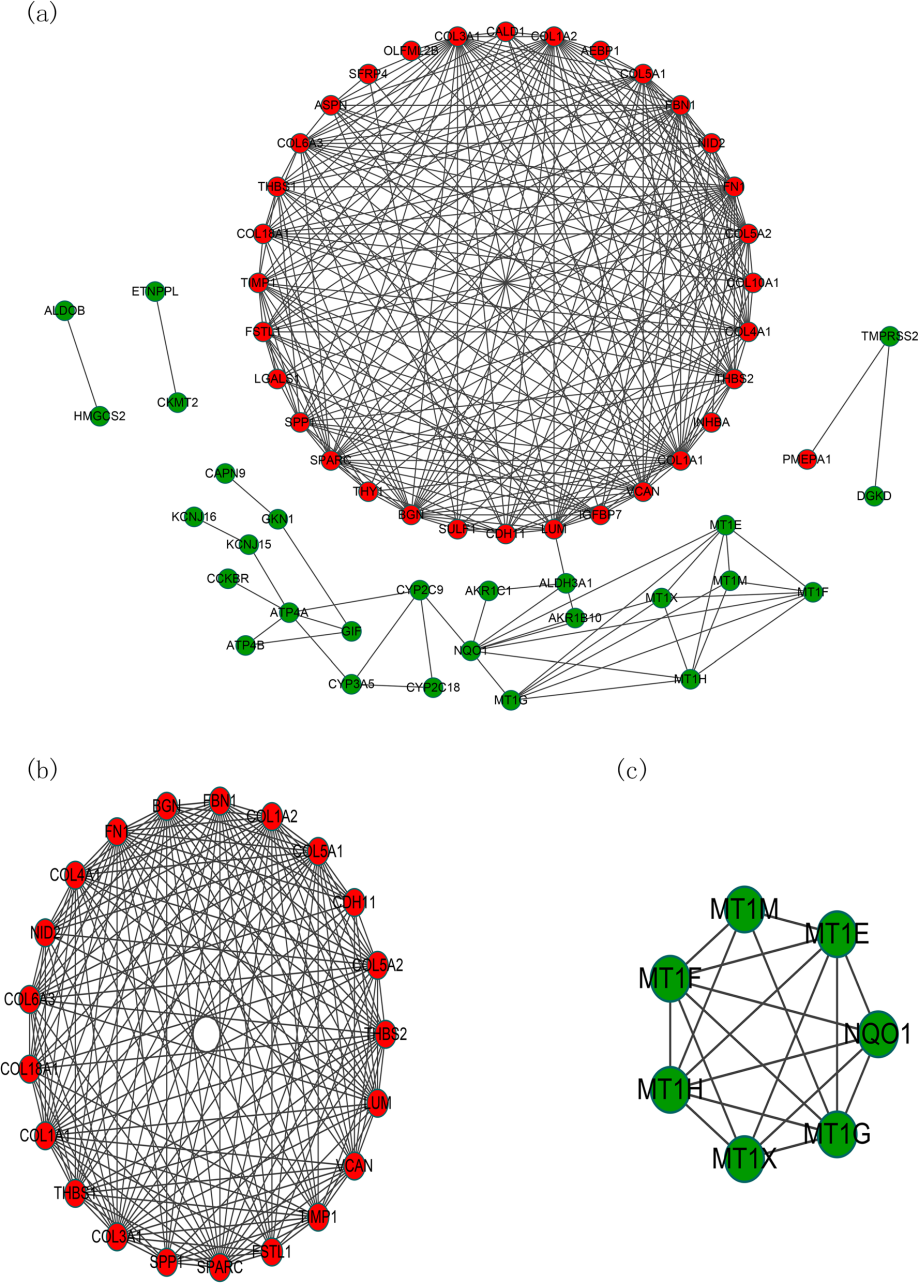
Establishment of PPI network and modules analysis. **a** Entire PPI network. **b** PPI network of module 1. **c** PPI network of module 2

To further explore the interaction between these 83 DEGs, the STRING database was used to construct PPI networks, and the resulting PPI networks were constructed using Cytoscape (Fig. 2a). Then, using MCODE, two key modules were identified from the whole network (Fig. 2 b and c). There were 21 nodes and 177 edges in module 1. In module 2, there were seven nodes and 19 edges. In order to identify hub genes, two algorithms (DMNC and MCC) of the cytoHubba app in the Cytoscape software were used. The top 10 hub genes based on the two methods were screened, and there were four mutual hub genes from the two methods: COL5A1, FBN1, SPARC, and LUM.

### Validation and survival analysis based on TCGA database

**Fig. 3:**
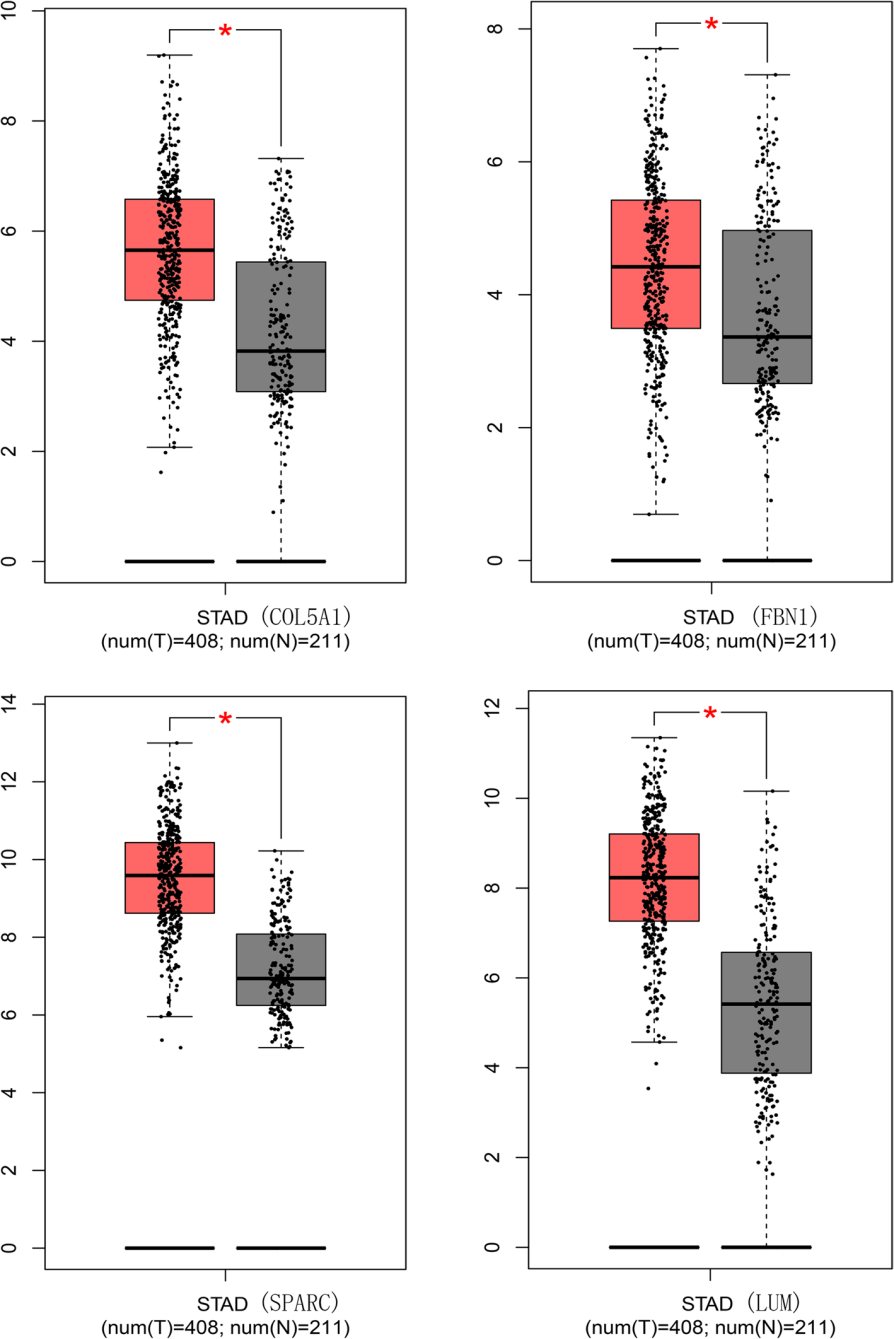
Box plots of four hub gene expressions in TCGA database

**Fig. 4:**
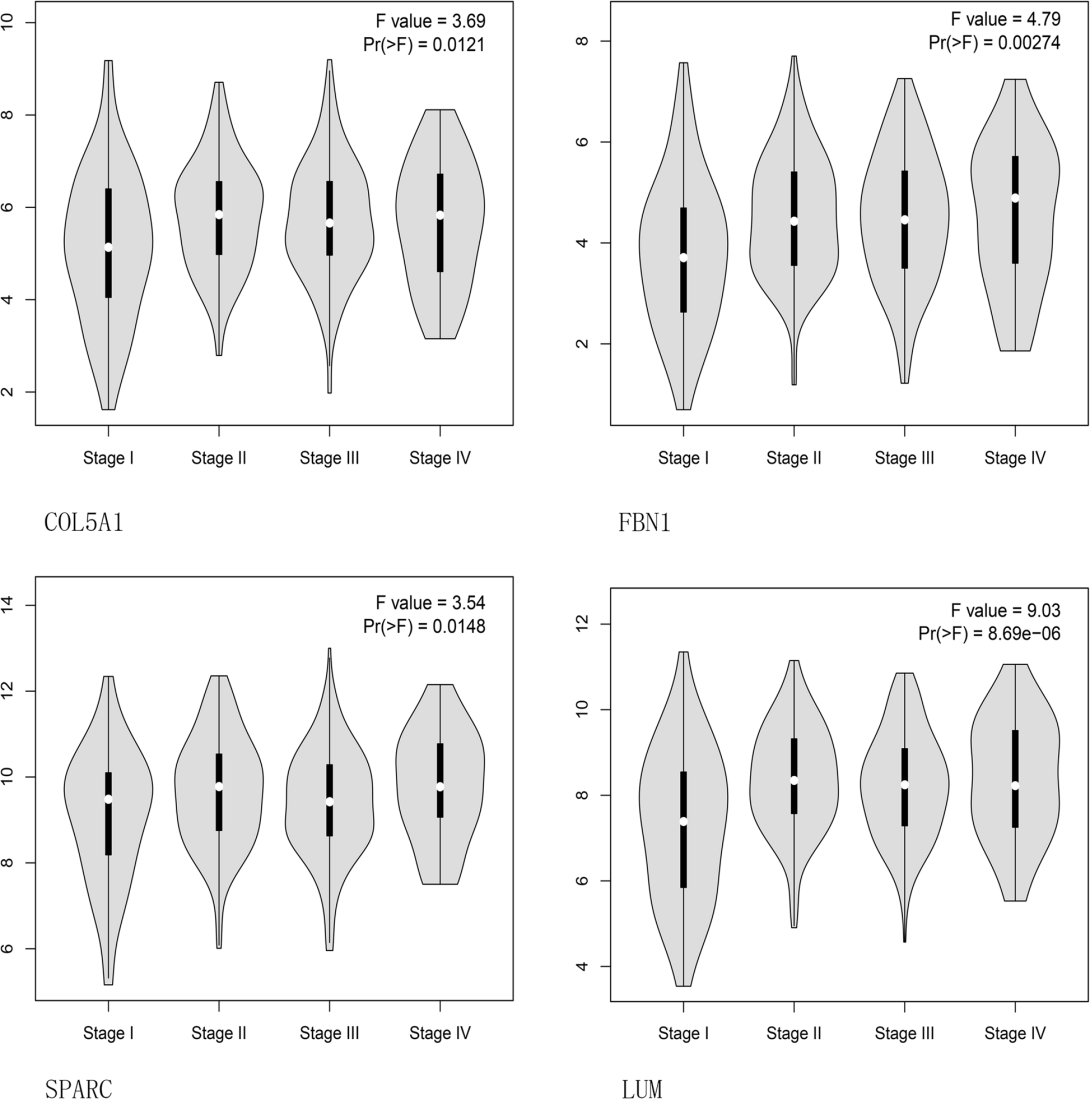
Plots of four hub gene expressions in different stages of GC

**Fig. 5:**
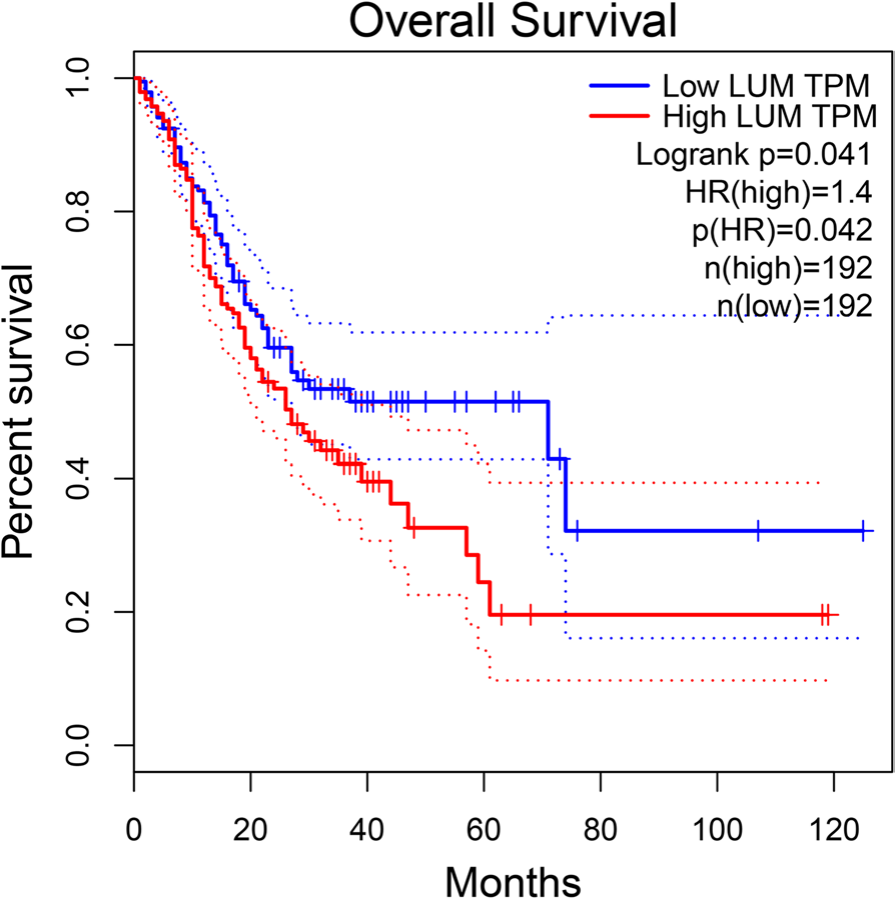
Kaplan–Meier survival analysis of LUM

To validate the results given above, the gene expression profiles of these four hub genes from TCGA database were used. GEPIA was used to visualize and analyze integration of TCGA database. These hub genes were significantly differentially expressed (*P* < 0.01), which was consistent with the results from the GEO data sets (Fig. 3). These hub genes were differentially expressed across various stages of GC (Fig. 4). Only LUM was significantly closely correlated with the overall survival of GC patients (log-rank *P* = 0.041; Fig. 5).

## Discussion

In this study, we integrated three microarray expression profiles from GEO and identified 83 DEGs between GC and normal gastric tissues, including 41 upregulated and 42 downregulated genes. Functional enrichment and KEGG pathway analysis showed that the DEGs primarily enriched in ECM organization, ECM-receptor interaction, and cell adhesion pathways. Our results suggested that these DEGs may play important role in the progression of GC.

ECM organization and ECM-receptor interaction have been proven to be an important part of tumorigenesis and development [16]. Genes encoding proteins that mediate ECM remodeling were upregulated in patients with prostate, lung, and gastric cancers [17]. Collagens are the most abundant ECM components, and they can regulate the physical and biochemical properties of the tumor microenvironment, which modulate cancer cell polarity, migration, and signaling [18, 19]. Cell adhesion is a key mediator of cancer progression and facilitates cancer metastatic dissemination. Many cell adhesion molecules within the tumor microenvironment are changed, and these changes alter the ability of tumor cells to interact with other cells and proteins of the ECM [20].

We also identified four major hub genes through the establishment of the PPI network by the STRING database and modules analysis, namely, COL5A1, FBN1, SPARC, and LUM. Subsequent survival analysis of these genes revealed that one of these four upregulated genes was closely related to the poor prognosis of GC patients.

The collagen type 5 α-1 chain (COL5A1) encodes an alpha chain for one of the low-abundance fibrillar collagens. In the research on ovarian cancer, COL5A1 is a poor outcome gene signature. Collagen remodeling might be a common biological process that contributes to poor overall survival [21]. Some studies have suggested COL5A1 is highly expressed at the mRNA and protein levels in breast cancer, and the patients with breast cancer with high COL5A1 expression have a reduced prognosis [22]. In GC, the COL family is a promising prognostic marker [23]. Fibrillin 1 (FBN1) is overexpressed in testicular germ cell tumors relative to nonneoplastic testicular tissue in patients with germ cell tumors, and it could be involved in germ cell neoplasia in situ development [24]. Silencing FBN1 could inhibit the cell proliferative, migratory, and invasive abilities of GC cells, while the influence of upregulated FBN1 expression showed the opposite effect [25]. Secreted protein acidic and rich in cysteine (SPARC) is a matricellular protein modulating cell-matrix interactions and has been found upregulated in colorectal tumor stroma. High SPARC was associated with better disease outcome in stage 2 colorectal cancer, but not in stage 3 colorectal cancer. It may play different roles in different development stages of colorectal cancer [26]. However, SPARC is upregulated in gastric cancer tissues relative to normal gastric tissues. High SPARC expression is associated with worse outcomes than negative and low SPARC expression, and SPARC is a potential marker for poor gastric cancer prognosis [27].

Lumican (LUM) is a protein-coding gene that encodes a member of the small leucine-rich proteoglycan (SLRP) family, which includes decorin, biglycan, fibromodulin, keratocan, epiphycan, and osteoglycin [28]. In recent years, an increasing number of experimental data has come to show that LUM is expressed in many kinds of tumors, including colorectal, prostate, lung, and pancreatic cancer [29–32]. The role of LUM in cancer varies according to the type of tumor. LUM is highly expressed in bladder cancer tissues and cell lines, and increased LUM expression is associated with the histological grade and the T/N stage of bladder tumors. The in vitro and in vivo data further indicate that low expression of LUM can inhibit the growth and migration of bladder cancer cells by inactivating MAPK signaling [33]. In node-negative invasive breast cancer, low lumican expression has a worse survival [34].

We provide reliable molecular biomarkers for therapy and prognosis of GC based on integrated bioinformatics analysis, including GO, KEGG pathway enrichment, PPI network, module analysis, and TCGA database, particularly when two algorithms are used to identify hub genes. However, our study has a number of limitations that should be considered. First, although we used the TCGA database to valid the results of GEO, molecular experiments are urgently needed to verify. Although we integrated three microarray data, large sample size is needed to validate the results. Second, we compared the paired GC tissues and their matched adjacent tissues. Many details were not taken into account, including histological type, grade of GC, and the distance from adjacent tissue to cancerous tissue. All of these may affect the expression of DEGs. Finally, in order to reduce the number of false-positive DEGs, we obtained co-expressed DEGs in three datasets. In this way, many important genes may have been lost.

## Conclusions

We screened DEGs associated with GC by integrated bioinformatics analysis and found one potential biomarker that may be involved in the progress of GC. This hub gene may serve as a guide for further molecular biological experiments.

## Data Availability

The data that support the findings of this study are available from the Gene Expression Omnibus (http://www.ncbi.nlm.nih.gov/geo/) and the Gene Expression Profiling Interactive Analysis (GEPIA) database (http://gepia.cancer-pku.cn/detail.php).

## Abbreviations

GC: Gastric cancer
GEO: Gene Expression Omnibus
GO: Gene ontology
KEGG: Kyoto Encyclopedia of Genes and Genomes
FC: Fold change
DAVID: Database for annotation, visualization, and integrated discovery
DEGs: Differentially expressed genes
PPI: Protein-protein interaction
TCGA: The Cancer Genome Atlas
ECM: Extracellular matrix
MCODE: Molecular Complex Detection
STRING: Search Tool for the Retrieval of Interacting Genes
DMNC: Density of maximum neighborhood component
MCC: Maximal clique centrality
GEPIA: Gene Expression Profiling Interactive Analysis
BP: Biological processes
CC: Cell component
MF: Molecular function
